# Genome Sequencing of *Streptomyces atratus* SCSIOZH16 and Activation Production of Nocardamine via Metabolic Engineering

**DOI:** 10.3389/fmicb.2018.01269

**Published:** 2018-06-13

**Authors:** Yan Li, Chunyan Zhang, Chengxiong Liu, Jianhua Ju, Junying Ma

**Affiliations:** ^1^CAS Key Laboratory of Tropical Marine Bio-resources and Ecology, Guangdong Key Laboratory of Marine Materia Medica, RNAM Center for Marine Microbiology, South China Sea Institute of Oceanology, Chinese Academy of Sciences, Guangzhou, China; ^2^College of Life Sciences, University of Chinese Academy of Sciences, Beijing, China; ^3^Hubei Key Laboratory of Natural Products Research and Development, College of Biological and Pharmaceutical Sciences, China Three Gorges University, Yichang, China

**Keywords:** *Streptomyces atratus* ZH16, in-frame deletion, metabolic engineering, siderophore, nocardamine

## Abstract

The *Actinomycetes* are metabolically flexible microorganisms capable of producing a wide range of interesting compounds, including but by no means limited to, siderophores which have high affinity for ferric iron. In this study, we report the complete genome sequence of marine-derived *Streptomyces atratus* ZH16 and the activation of an embedded siderophore gene cluster via the application of metabolic engineering methods. The *S. atratus* ZH16 genome reveals that this strain has the potential to produce 26 categories of natural products (NPs) barring the ilamycins. Our activation studies revealed *S. atratus* SCSIO ZH16 to be a promising source of the production of nocardamine-type (desferrioxamine) compounds which are important in treating acute iron intoxication and performing ecological remediation. We conclude that metabolic engineering provides a highly effective strategy by which to discover drug-like compounds and new NPs in the genomic era.

## Introduction

Microbially produced natural products (NPs) are one of the most important classes of compounds known to mankind having a vast assortment of applications in medical and agricultural sectors (Bérdy, 2005). It has been estimated that most major classes of antibiotics and over 70% of anti-cancer small molecule therapeutics are microbial NPs, their derivatives, or related congeners/analogs (Newman and Cragg, 2016). With the recently noted rise in resistance to antibiotics and cancer chemotherapeutics, it has become increasingly obvious that novel bioactive NPs are urgently needed to ensure the success of new drug discovery and development initiatives. Genomics and metabolomics have played central roles in ensuring that these needs get met.

Over the last two decades significantly improved technologies for genome sequencing have made it much easier to sequence full microbial genomes. Additionally, studies of microbial genomes have made clear that many microorganisms have far greater potential to produce specialized metabolites than previously thought (Rutledge and Challis, 2015). It has been estimated that the genomes of species from *Streptomyces* family members, the largest bacterial genus house on average, about 20 biosynthetic gene clusters (BGCs) coding for NPs (Nett et al., 2009). However, over 80% of these gene clusters are typically orphaned under normal laboratory culturing conditions (Nett et al., 2009; Baltz, 2017). Consequently, metabolic engineering and genome mining methods have increasingly been applied to discover secondary metabolites whose corresponding BGCs are normally silent; such BGCs are also sometimes considered “orphan BGCs” to convey the absence of a correlatable NP/s (Zerikly and Challis, 2009; O’Connor, 2015; Baltz, 2016).

Microbial siderophores biosynthesis can generally be classified into two main pathways: non-ribosomal-peptide synthetase (NRPS)-dependent and siderophore synthetase super-family (Barry and Challis, 2009); both pathways are exploited by a range of genera belonging to the *Actinomycetes* (Wang et al., 2014). Many siderophores are NRPS dependent family NPs, such as griseobactin (Patzer and Braun, 2010), coelichelin (Challis and Ravel, 2000), oxachelin (Sontag et al., 2006), as well as tsukubachelin (Kodani et al., 2011, 2013), peucechelin (Kodani et al., 2015), and chlorocatechelins (Kishimoto et al., 2014). Nocardamine is the representative one of the siderophore synthetase super-family (Stoll et al., 1951; Hossain et al., 1983; Ueki et al., 2009). Nocardamines (also called desferrioxamines), composed of alternating dicarboxylic acid and diamine units, originally isolated as antibacterial metabolites from a *Nocardia* strain (Stoll et al., 1951). The BGC responsible for desferrioxamines G_1_ and E in *Streptomyces coelicolor* A3(2) was investigated by Barona-Gómez et al. (2004). Among their findings was that the *des* operon contained a subset of four genes coding for the production of various desferrioxamines (Barona-Gómez et al., 2004); their production was found to be regulated by both iron concentrations and by an iron-dependent regulatory protein-IdeR (Günter et al., 1993; Ueki et al., 2009).

*Streptomyces atratus* SCSIO ZH16 is a deep sea-derived *Streptomyces* that predominantly produces ilamycins under standard laboratory conditions; the biosynthesis of ilamycins has been elucidated in our previous studies (Ma et al., 2017). By applying a combination of Frameplot 3.0 beta (Ishikawa and Hotta, 1999) and AntiSMASH 3.0 (Weber et al., 2013), two online software systems, we were able to predict that up to 26 BGCs are housed within the genome of *S. atratus* SCSIO ZH16; we envisioned that the majority of these are orphan clusters. Accordingly, we applied metabolic engineering methods to activate these putative orphan/silent clusters *en route* to the production of new compounds with potential applications in drug discovery and bioremediation. Here we report: (i) the complete genome sequence of *S. atratus* SCSIO ZH16 as well as a comparative analysis to get further insights into genetic elements involved in biosynthesis of NPs, (ii) the construction of in-frame deletion mutant *S. atratus* SCSIO ZH16S and *S. atratus* SCSIO ZH16NS, and (iii) the identification and structural characterization of nocardamine. Our study highlights the enabling power of metabolic engineering to generate new NPs encoded by orphan gene clusters and also validates the engineered *S. atratus* ZH16NS as a promising nocardamine-based siderophore producer.

## Experimental Section

### General Experimental Section

All bacteria, plasmids and primers used in this work are listed in Supplementary Tables S1, S2. The antibiotics and reagents were purchased from Sangon Biotech Co., Ltd. (Shanghai, China), the PCR polymerase and related reagents were purchased from Takara Biotechnology Co., Ltd. (Dalian, China), gel recycle and PCR recycle kits were purchased from Omega Bio-tek Inc. (Norcross, GA, United States). All solvents were analytical or chromatographic grade and purchased from Guangzhou Chemical Reagent Factory (Guangzhou, China) and Thermo Fisher Scientific Inc. (Waltham, MA, United States).

Column chromatography (CC) was carried out using normal phase silica gel (100–200 mesh, Jiangyou, China) and reverse phase C18 silica gel (40–63 μm, Merck, Germany). Medium-pressure liquid chromatography was performed with a CHEETAH 100 automatic flash chromatography system (Bonna-Agela, China) with an ODS-A flash column (S-50 μm, 12 nm; 100 mm × 20 mm, YMC, Japan). Semi-preparative HPLC was carried out using an Agilent 1260 liquid chromatography system with diode array detector (DAD) (Agilent, United States) and YMC-Pack ODS-A column (250 mm × 20 mm, 5 mm, YMC, Japan). NMR spectra were performed with an Advance 700 MHz spectrometer (Bruker, Germany). High-resolution mass spectral data was obtained from a MaXis quadrupole-time-of-flight mass spectrometer (Bruker, Germany). All the gene amplification was performed with Eppendorf mastercycler pro PCR equipment (Eppendorf, Germany).

### Genome Sequencing Bioinformatic Analysis

The collection, identification, and genome sequencing of *S. atratus* SCSIO ZH16 has been previously described (Ma et al., 2017). BGCs and their related ORFs were analyzed by antiSMASH 3.0^1^ and Frame Plot 3.0 beta ^2^, respectively. Furthermore, functional gene annotations and sequence alignments were carried out using Basic Local Alignment Search Tool^3^.

### In-Frame Deletion of Ilamycin Genes

To obtain NPs encoded by other orphan/silent gene clusters using metabolic engineering methods in *S. atratus* SCSIO ZH16, genetic engineering mutants with clean metabolic background were constructed. IlaS has been identified as a large non-ribosomal peptide synthetase responsible for the incorporation of amino acid building blocks to form the full-length heptapeptide. IlaN encoding a cytochrome P450 monooxygenase and IlaO encoding a prenyltransferase were involved in the biosynthesis of L-3-nitrotyrosine and *N*-(1,1-dimethyl-1-allyl)-tryptophan building blocks, respectively. In-frame gene deletions were achieved by following the REDIRECT protocol (Gust et al., 2003). The *S. atratus* SCSIO ZH16 genomic cosmid library was constructed as previously reported (Ma et al., 2017). The apramycin resistance gene *oriT*/*aac(3)*IV fragment was obtained by using specific primers that contain additional *Spe*I restriction sites, and used to replace the target genes in the cosmids 2-10E or 4-07H (Supplementary Table S2). Restriction digests of mutant cosmids with *Spe*I and subsequent relegation predictably abolished the apramycin resistance gene *oriT*/*aac(3)*IV fragment. The second round of PCR-targeting was performed to replace the kanamycin resistance gene on SuperCos I with another apramycin resistance gene *oriT*/*aac(3)*IV fragment obtained by a primer pair ARK (Zhang et al., 2013). The constructed mutant cosmids were introduced into non-methylating *Escherichia coli* ET12567/pUZ8002 and then transferred into *S. atratus* SCSIO ZH16 by conjugation. Because the strain was sensitive to apramycin, exconjugants were grown on solid apramycin containing ISP-4 medium to select for the chromosomal integration of the inactivation constructs. To ensure loss of the target gene from the chromosome, exconjugants were replica-plated once onto antibiotic-free ISP-2 plates. Single colonies were again replica-plated onto apramycin-containing ISP-2 plates and antibiotic-free ISP-2 plates. Apramycin-sensitive clones were evaluated by PCR to ensure proper generation of the desired mutant clones. Two in-frame deletion mutants, *S. atratus* SCSIO ZH16S and *S. atratus* SCSIO ZH16NS, were obtained using this method.

### Fermentation and Isolation

The *S. atratus* SCSIO Zh16 wild-type, *S. atratus* SCSIO ZH16S, and *S. atratus* SCSIO Zh16NS mutant strains were cultured in 250 mL flasks containing 50 mL Am2ab liquid media consisting of 0.5% soluble starch, 2% glucose, 0.2% yeast extract, 0.2% peptone, 0.5% soybean meal, 0.05% MgSO_4_.7H_2_O, 0.05% KH_2_PO_4_, 0.4% NaCl, 0.2% CaCO_3_, and 3% crude sea salt. Then they were incubated at 28°C on a rotary shaker at 200 rpm for 7 days. Fermentation broths were extracted by 80 mL butanone and the solvent was removed under reduced pressure to give crude extracts that were dissolved in 0.6 mL methanol. Each extract was ultimately subjected to HPLC analysis. The HPLC analysis was carried out with a reversed phase column SB-C18, 5 μM, 4.6 × 150 mm (Aglient, United States) with UV detection at 210 and 285 nm under the following program: solvent system (solvent A, water supplemented with 0.1% trifluoroacetic acid; solvent B, acetonitrile supplemented with 0.1% trifluoroacetic acid); 2–98% solvent B (linear gradient, 0–30 min), 98% solvent B (30–35 min), 98–2% solvent B (35.0–35.1 min), 2% solvent B (35.1–40 min); flow rate was set at 1 ml/min.

The large-scale fermentation of *S. atratus* SCSIO ZH16NS was performed using a two-stage fermentation process. The spores grown on ISP-2 supplemented with 3.0% crude sea salt plates were incubated in a total of 63 flasks (250 mL volume) containing 50 mL Am2ab liquid media at 28°C on a rotary shaker at 200 rpm for 60 h. The 25 mL seed cultures were then transferred into 1 L flasks containing 200 mL Am2ab medium with supplemental 3.0% XAD-16 resin. The resin-containing mixtures were then cultured at 28°C on a rotary shaker at 200 rpm for 8 days. Aliquots (10 mL) were removed from each flask on a daily basis and analyzed by HPLC. After 8 days of growth fermentation broths (28 L) were centrifuged to ensure separation of supernatant, mycelium and resin which were extracted sequentially with twofolds volume butanone (3x), acetone (3x), and EtOH (3x), respectively.

The following HPLC analyses, all extracts (butanone, acetone, and EtOH) were combined, solvent removed *in vacuo* and the remaining extracts subjected to silica gel CC using gradient elution with a CHCl_3_-MeOH mixture (100:0, 98:2-1, 98:2-2, 96:4-1, 96:4-2, 94:6-1, 94:6-1, 92:8, 9:1,85:15, 8:2,1:1, 0:100) to give 13 fractions (Fr.A1–Fr.A13). Fr.A7 was purified by MPLC with ODS column, which was eluted from 0 to 60% solvent B (A: H_2_O, B: CH_3_CN) over the course of 60 min to obtain 14 fractions (Fr.J1–Fr.J14). Fr.J2-Fr.J3 were combined and further purified by Sephadex LH-20 chromatography eluted by MeOH and semi-preparative HPLC eluted from 5 to 20% solvent B over 10 min and 20% solvent B over 5 min at a flow rate of 2.5 mL/min using a detection wavelength of 210 nm.

### Structural Elucidation

Nocardamine: white acicular crystal, ^1^H and ^13^C NMR data (**Table 1**). HRESIMS: m/z 601.3556 ([M+H]^+^ calcd 601.3553), m/z 623.3402 ([M+Na]^+^ calcd 623.3384).

**Table 1:** The ^1^H NMR and ^13^C NMR data of nocardamine [In CDCl_3_ and MeOD (1:1), 700 MHz ^1^H NMR and 175 MHz^13^C NMR in *δ* ppm].

Position	δ_C_	δ_H_ (mult, *J* in Hz)
1, 12, 23	–	–
2,13, 24	174.5 CO	–
3, 14, 25	31.3 CH_2	2.51 (2H×3, t, *J* = 7.2)
4, 15, 26	28.5 CH_2	2.80 (2H× 3, t, *J* = 7.2)
5, 16, 27	173.9 CO	–
6, 17, 28	–	–
7, 18, 29	39.7 CH_2	3.20 (2H× 3, t, *J* = 6.5)
8, 19, 30	29.1 CH_2	1.54 (2H× 3, m)
9, 20, 31	23.9 CH_2	1.33 (2H× 3, m)
10, 21, 32	26.5 CH_2	1.65 (2H× 3, m)
11, 22, 33	48.1 CH_2	3.63 (2H× 3, t, *J* = 6.5)

## Results and Discussion

### Genome Sequencing and Annotation of *Streptomyces atratus* SCSIO ZH16

**FIGURE 1:**
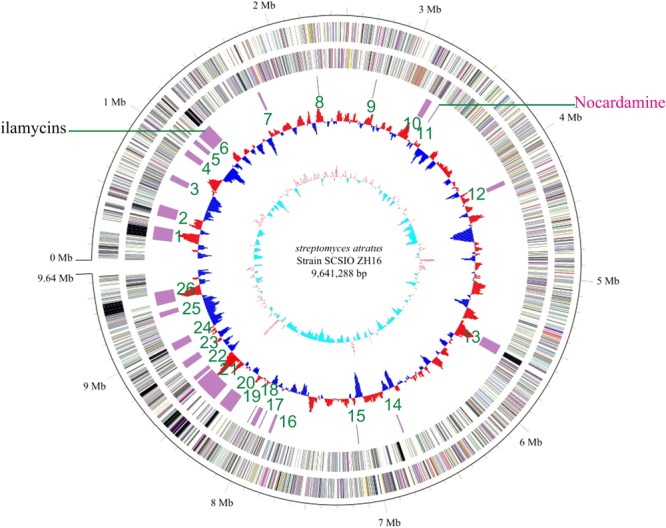
The complete genome of *Streptomyces atratus* SCSIO ZH16. The five circles (outer to inner) represent forward strand CDSs, reverse strand CDSs, nomenclature, and locations of predictive secondary metabolites generated using antiSMASH 3.0 software, GC content and GC skew. Putative nocardamine cluster herein referred to cluster *noc*.

Genome sequence information is playing a progressively more important role in NPs discovery as well as studies to elucidate NP biogenesis. Many antibiotic producing strains have been sequenced and examinations of their genomic data have revealed their full biosynthetic potentials which are often far exceeded initial expectations. To better understand the full secondary metabolic potential of *S. atratus* SCSIO ZH16, its genome was sequenced using a combination of 2nd-generation 454 and Illumina HiSeq 4000 sequencing technologies and 3rd-generation PacBio sequencing technology at Shanghai Biozeron Co., Ltd. The assembled genome, along with elucidation of its GC content enabled its classification as a *Streptomyces.* The *S. atratus* SCSIO ZH16 genome is 9,641,288 bp long and consists of a linear chromosome with an average GC content of 69.5% (**Figure 1**). The genome contains 9245 coding sequences, 18 rRNA genes and 69 tRNA genes for transfer of all 20 amino acids (Supplementary Table S3). The genome sequence of *S. atratus* SCSIO ZH16 has been deposited in the Genbank database with the accession number of CP027306.

To elucidate the gene clusters encoded in its genome, the assembled genome sequence was subjected to analysis for secondary metabolite BGCs using online antiSMASH software (see footnote text 1) (Weber et al., 2013) and Frameplot 3.0 beta (Ishikawa and Hotta, 1999). These analyses revealed 26 gene clusters within the *S. atratus* SCSIO ZH16 genome, including six NRPS, four PKS (Type I, Type II, and Type III), four hybrid PKS-NRPS, four terpene, three bacteriocin, two siderophore and three other categories BGCs (Supplementary Table S4) indicating the great potential of the strain to produce an array of secondary metabolites. Significantly, only one gene cluster responsible for ilamycin biosynthesis has been characterized and elucidated from the wild-type strain (Ma et al., 2017). This strain will serve as a target for further genome mining of secondary metabolite BGCs.

### Construction of Ila Gene Cluster In-Grame Deletion Mutants and the Discovery of a New Peak in the Genetic Engineered Mutant

**FIGURE 2:**
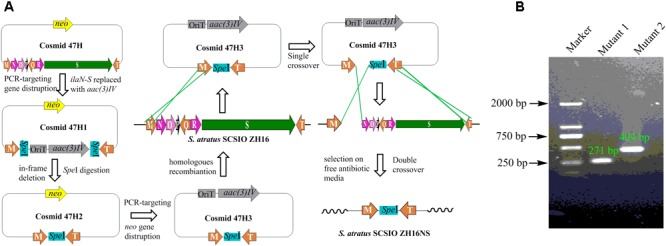
Construction the in-frame gene deletion mutants of *S. atratus* SCSIO ZH16. **(A)** Depiction of constructing in-frame deletion mutant *S. atratus* SCSIO ZH16NS. The 8 kb fragment on cosmid 47H was replaced by the apramycin resistance gene *aac(3) IV* using λ-Red mediated recombination. The resistance marker was cut by *speI* digestion to afford a mutated cosmid47H2 harboring a *speI* sites. Then kanamycin resistance gene was replaced by the apramycin resistance gene, leading to mutated cosmid 47H3. After the conjugation of mutated cosmid 47H3 with *S. atratus* SCSIO ZH16, exconjugant colonies were screened for single crossover mutants by using apramycin resistance. Single recombination colonies were grown on free antibiotic plates to promote double crossover mutant leading to the loss of the target genes. The construction process of in-frame deletion mutant, *S. atratus* SCSIO ZH16S, was the same with that of *S. atratus* SCSIO ZH16NS. **(B)** DNA gel electrophoresis of PCR products obtained with in-frame mutant strain (M: maker, Mutant1: *S. atratus* SCSIO ZH16S, Mutant2: *S. atratus* SCSIO ZH16NS).

**FIGURE 3:**
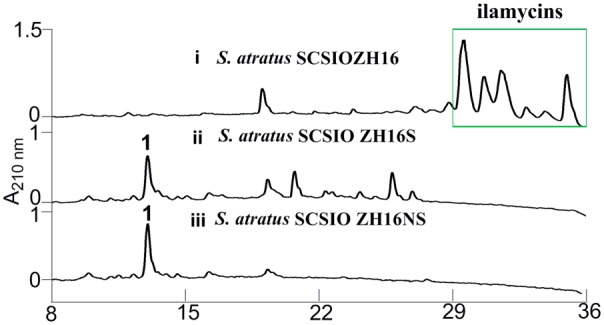
HPLC analyses of metabolite profile for different *S. atratus* strains. (i) *S. atratus* SCSIO ZH16 wild-type; (ii) *S. atratus* SCSIO ZH16S in-frame mutant; (iii) *S. atratus* SCSIO ZH16NS in-frame mutant. 1 is nocardamine.

Secondary metabolites may be overlooked due to low production levels, a large metabolic background or unpropitious culture conditions (Scherlach and Hertweck, 2009); indeed such considerations have inspired the term “orphan” instead of “silent” BGCs – it is not that a BGC is completely inactive, rather its product simply has not been identified. In our previous report, we have identified IlaS as a large non-ribosomal peptide synthetase responsible for the incorporation of amino acid building blocks to form the full-length heptapeptide of ilamycins (Ma et al., 2017). To enable the production of potential secondary metabolites from *S. atratus* SCSIO ZH16, an in-frame deletion mutant *S. atratus* SCSIO ZH16S (**Figure 2**), devoid of the 1.5 kb *ilaS* gene was constructed to abolish production of the ilamycins, a predominant product of wild-type *S. atratus* SCSIO ZH16 (**Figure 3**, i). Subsequent HPLC analyses revealed that, indeed, ilamycins biosynthesis was abolished upon deletion of *ilaS* (**Figure 3**, ii). However, the ilamycins precursor *N*-(1,1-dimethyl-1-allyl)-tryptophan generated by IlaO from tryptophan was identified in the fermentation extracts of the Δ*ilaS* mutant strain. The generation of a clean background strain for genome mining was not possible. We have demonstrated that IlaN (a cytochrome P450 monooxygenase) is involved in the biosynthesis of L-3-nitrotyrosine building block, and that IlaO (a prenyltransferase) is responsible for the biosynthesis of *N*-(1,1-dimethyl-1-allyl)-tryptophan building block (Ma et al., 2017). Therefore, we sought to further reduce the metabolic background of a genetically engineered *S. atratus* SCSIO ZH16 by carrying out further gene deletions. The in-frame deletion mutant *S. atratus* SCSIO ZH16NS was generated via deletion of an 8-kb fragment spanning from *ilaN* to *ilaS* (**Figure 2**). This mutant strain failed to produce ilamycins as well as the previously noted tryptophan-derived ilamycin precursor (**Figure 3**, iii) and consequently served as an excellent starting strain for the genome mining of *S. atratus* SCSIO ZH16.

In order to analyze the metabolomic differences between genetic engineered mutants and their wild type predecessor *S. atratus* SCSIO ZH16, HPLC chromatograms of extracts from the fermentations of the three strains were carefully compared. Notably, a new peak was identified uniquely in the *S. atratus* SCSIO ZH16S and *S. atratus* SCSIO ZH16NS mutants. HRESIMS analysis of the signal generating species revealed a low molecular weight NP with [M+H]^+^ = 601.3556, [M+Na]^+^ of 623.3402 (Supplementary Figure S1); on the basis of these data this mutant specific compound was assigned a molecular formula of C_27_H_48_N_6_O_9_.

### Fermentation, Isolation, and Structural Elucidation of Nocardamine From *S. atratus* SCSIO ZH16NS

**FIGURE 4:**
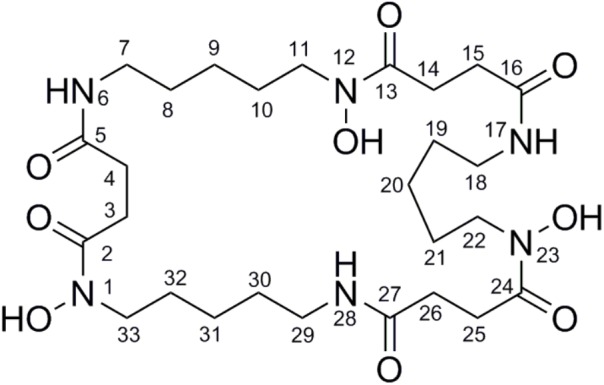
The structure with the numbered C and N atom has been submitted to our journal system to replace this one.

To isolate and elucidate the structure of the newly generated NP, large-scale fermentation of *S. atratus* SCSIO ZH16NS was carried out by using 28 L of Am2ab liquid media with a two-step fermentation process as previously reported (Ma et al., 2017). A compound peak retention time of 13.2 min was apparent when using detection at 210 nm; this signal correlated perfectly to the species originally identified on analytical scale fermentations/analysis with *S. atratus* SCSIO ZH16NS. After several rounds of silica column isolation and medium pressure preparative HPLC, 13.3 mg of analytically pure compound was obtained. For elucidating the structure of the purified compound, the NMR analysis was carried out. The NMR data revealed that ^1^H and ^13^C NMR data (**Table 1** and Supplementary Figures S2, S3) which were consistent with the nocardamine spectral data reported in the literature (Yuan et al., 2010). So the compound was identified as nocardamine (also called desferrioxamine E) (**Figure 4**).

### Bioinformatic Analysis of the Nocardamine Gene Cluster and the Proposed Biosynthetic Pathway of Nocardamine in *S. atratus* SCSIO ZH16

**FIGURE 5:**
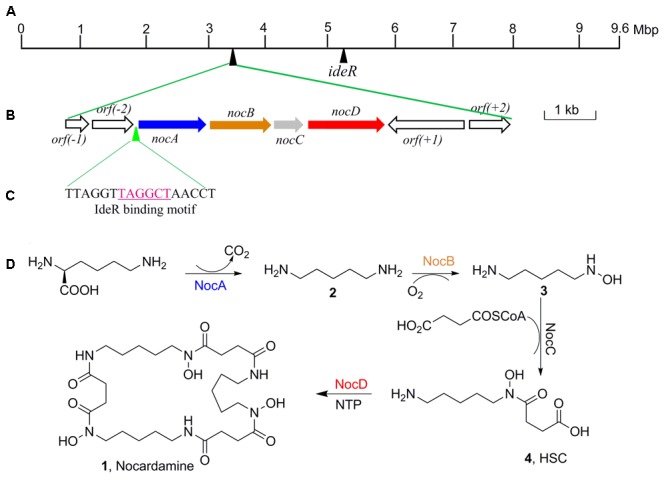
The organization of nocardamine biosynthetic gene cluster and the IdeR binding motif sequence. **(A)** The locations of nocardamine biosynthetic gene cluster and the *ideR* gene. **(B)** The organization of nocardamine biosynthetic gene cluster. **(C)** The magenta sequence was the binding motif of IdeR. **(D)** The proposed biosynthetic pathway of nocardamine.

Further to identify the gene cluster likely responsible for nocardamine biosynthesis, all clusters encoded in the genome of *S. atratus* SCSIO ZH16 were surveyed. Of the 26 unidentified gene clusters, there are two siderophore gene clusters, while cluster 11 showed a high degree of similarity to the previously published desferrioxamine B BGC (Barona-Gómez et al., 2004; Ueki et al., 2009). Accordingly, we assigned this cluster as the nocardamine (desferrioxamine E) BGC. The detailed annotation of the nocardamine gene cluster in *S. atratus* SCSIO ZH16 was postulated and found to contain a *noc* operon with a subset of four genes, enough to code for the biosynthesis of nocardamine (desferrioxamine E). More rigorous BLAST analysis showed that the four genes *nocABCD* in *S. atratus* SCSIO ZH16 encode for pyridoxal decarboxylase (*nocA* gene), putative monooxygenase (*nocB* gene), *N*-acetyltransferase (*nocC* gene), and *IucA*/*IucC* family siderophore biosynthesis protein (*nocD* gene) having 83, 77, 60, 73% identity with *desA, desB, desC, desD* in *S. coelicolor* M145, respectively (**Table 2**). The BGC for desferrioxamine has been reported or characterized in another two *Streptomyces* strains, *Streptomyces avermitilis* K139 (Ueki et al., 2009), and *S. pristinaespiralis* HCCB10218 (Li et al., 2015). Gene functions and organization were identical in each desferrioxamine BGC. The location and organization of the nocardamine gene cluster in the chromosome of *S. atratus* ZH16 were shown in **Figures 5A,B**.

**Table 2:** Deduced function of individual *orfs* within the *noc* cluster from *Streptomyces atratus* ZH16.

Protein	Size (aa)	Proposed function	Protein homologue,	Protein homologue,
			origin^a^, ID/SI (%)	origin^b^, ID/SI (%)
ORF(-2)	119	Hypothetical protein	–	–
ORF(-1)	283	Siderophore-interacting protein	–	–
NocA	493	L-2,4-diaminobutyrate decarboxylase	DesA, 83/89	SGR_4750, 88/93
NocB	425	Putative monooxygenase	DesB, 77/86	AlcA, 85/91
NocC	194	*N*-acetyltransferase	DesC, 60/70	AlcB, 76/82
NocD	592	IucA/IucC family sidero-phore biosynthesis protein	DesD, 73/82	AlcC, 84/90
ORF(+1)	553	Hexosaminidase	–	–
ORF(+2)	308	Tat pathway signal sequence domain protein	–	–

*ID/SI: Identity/Similarity; ^*a*^*Streptomyces coelicolor* M145; ^*b*^*Streptomyces griseus* subsp. *griseus* NBRC 13350.*

Based on the proposed functions of the genes in *noc* gene cluster, the biosynthetic pathway of nocardamine was proposed as follows: firstly, L-lysine was decarboxylated to yield cadaverine (**2**) by L-2,4-diaminobutyrate decarboxylase encoded by *nocA*. Secondly, cadaverine is hydroxylated at an amino group by monooxygenase encoded by *nocB*, to form *N*-hydroxy-cadaverine (**3**). Then, *N*-hydroxy-cadaverine was condensed with a succinyl-CoA to generate the *N*-hydroxy-*N*-succinylcadaverine (HSC, **4**). Finally, three HSC units were catalyzed to form nocardamine by LucA/Iuc family siderophore biosynthesis protein encoded by *nocD*. The proposed biosynthetic pathway of nocardamine in *S. atratus* SCSIO ZH16 was shown in **Figure 5D**.

### The Possible Regulation of Nocardamine Production in *S. atratus* SCSIO ZH16

The production of nocardamine is carefully controlled by a regulatory protein termed iron-dependent regulatory protein (IdeR) (Günter et al., 1993). To investigate whether the production of nocardamine is also regulated by an IdeR ortholog, three *ideR* genes from *Streptomyces davawensis* strain JCM 4913, *Mycobacterium tuberculosis* H37Rv, and *S. coelicolor* A3(2) were used as probes for BLAST searching within the genome sequence of *S. atratus* SCSIO ZH16. BLAST results indicated that a *ideR* ortholog located at 5.1 Mbp of *S. atratus* SCSIO ZH16 chromosome genome has the highest identity (87%) and coverage (88%) with that from the *S. davawensis* strain JCM 4913, and has 86 and 80% identity with that from the *S. coelicolor* A3(2) and *M. tuberculosis* H37Rv, respectively. These results indicated that the production of nocardamine in *S. atratus* SCSIO ZH16 is likely regulated by *ideR* homologues. In order to further determine the binding motif of a putative IdeR regulator, the upstream sequence of the operon *nocABCD* was analyzed by comparison the binding motif to those previously reported in the literature (Günter et al., 1993; Ueki et al., 2009). These comparisons indicated that a 17 base pair sequence “TTAGGTTAGGCTAACCT” has the same IdeR binding motif reported in *S. avermitilis* K139 (Ueki et al., 2009), which is also a nocardamine producer. The regulatory function of IdeR in the production of nocardamine by *S. atratus* SCSIO ZH16 will be characterized in forthcoming publications. The location of the *ideR* gene in the chromosome of *S. atratus* SCSIO ZH16 was shown in **Figure 5A**.

## Conclusion

The deep sea-derived *S. atratus* SCSIO ZH16 has a linear genome chromosome whose average GC content is 69.5%. Twenty-six gene clusters housed in the genome endow the strain a potential target for genome mining of bioactive NPs. An orphan BGC (*noc*) coding for a siderophore has been activated via metabolic engineering; this entailed the construction and ensuing metabolite analyses of the mutant strains of *S. atratus* SCSIO ZH16S and *S. atratus* SCSIO ZH16NS. These mutants result from efforts to knock out selected portions of the well-studied ilamycin gene cluster. The in-frame deletion mutant of *S. atratus* SCSIO ZH16NS with a relative clean metabolic background, provides excellent opportunity to further mining other orphan gene cluster encoding NPs. The siderophore was structurally elucidated using a combination of HRESIMS and NMR analyses and shown to be previously reported nocardamine. By virtue of its excellent metal ion complexation abilities, nocardamine can be used as an iron carrier to relieve metal toxicity. This work highlights the notion that shifting metabolic flux of an NP producing wild-type strain away from the predominant product pathway may enable the production of new metabolites that, otherwise, are simply not attainable. We posit that nocardamine production (*noc* activation) is enabled at the expense of ilamycin biosynthesis by virtue of engineered shifting of the *S. atratus* metabolic flux. Studies to further dissect this system of engineered cluster activation in the unique marine-derived microbe will be published in due course.

## Author Contributions

JM and JJ designed the experiments and revised the manuscript. YL analyzed the BGC sequences, performed the mass fermentation of *S. atratus* SCSIO ZH16NS, isolated the compound, and prepared the draft manuscript. CZ constructed two in-frame deletion mutants of *S. atratus* SCSIO ZH16S and *S. atratus* SCSIO ZH16NS and wrote part of the draft manuscript. CL analyzed the NMR data. All authors reviewed the manuscript.

## Conflict of Interest Statement

The authors declare that the research was conducted in the absence of any commercial or financial relationships that could be construed as a potential conflict of interest. The reviewer JKS and handling Editor declared their shared affiliation.

## Footnotes

1 https://antismash.secondarymetabolites.org/#!/start

2 http://www0.nih.go.jp/~jun/cgi-bin/frameplot.pl

3 https://blast.ncbi.nlm.nih.gov/Blast.cgi
