# Supplementary material for: Genome Sequencing of Streptomyces atratus SCSIOZH16 and Activation Production of Nocardamine via Metabolic Engineering
Source: Front Microbiol. 2018 Jun 13;9:1269. doi: 10.3389/fmicb.2018.01269 (PMC6011815; doi:10.3389/fmicb.2018.01269)
Supplement: Supplementary file 1 [file Presentation_1.pdf]

Supplementary Material

Genome Sequencing of *Streptomyces atratus* SCSIOZH16 and Activated Production of Nocardamine via Metabolic Engineering

Yan Li [1,2], Chunyan Zhang [1,2], Chengxiong Liu [3] , Jianhua Ju,[1,2,]* Junying Ma [1,]*

[1] CAS Key Laboratory of Tropical Marine Bioresources and Ecology, Guangdong Key Laboratory of Marine Materia Medica, RNAM Center for Marine Microbiology, South China Sea Institute of Oceanology, Chinese Academy of Sciences, Guangzhou, China.

[2] University of Chinese Academy of Sciences, College of life sciences, Beijing, China.

[3] Hubei Key Laboratory of Natural Products Research and Development, College of Biological and Pharmaceutical Sciences, China Three Gorges University, Yichang, China.

* Correspondence
Jianhua Ju
jju@scsio.ac.cn;
Junying Ma
majunying@scsio.ac.cn

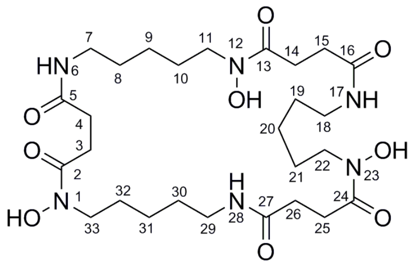

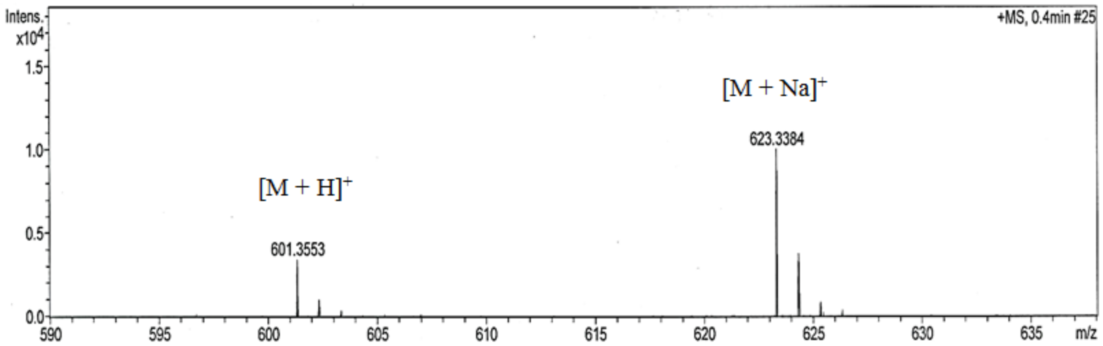

Meas. m/z	#	Ion Formula	Score	m/z	err [ppm]	err [mDa]	mSigma	rdb	e⁻ Conf	N-Rule
601.355333	1	C27H49N6O9	100.00	601.355554	-0.4	-0.2	12.5	6.5	even	ok
	2	C31H53O11	12.66	601.358239	-4.8	-2.9	23.8	5.5	even	ok
	3	C28H45N10O5	37.51	601.356891	2.6	1.6	24.1	11.5	even	ok
	4	C38H49O6	4.13	601.352366	-4.9	-3.0	61.4	14.5	even	ok
	5	C39H45N4O2	8.15	601.353703	-2.7	-1.6	73.1	19.5	even	ok
623.338399	1	C29H40N14NaO	52.18	623.340173	-2.8	-1.8	11.0	16.5	even	ok
	2	C31H52NaO11	46.81	623.340183	2.9	1.8	16.2	5.5	even	ok
	3	C28H44N10NaO5	100.00	623.338835	-0.7	-0.4	18.1	11.5	even	ok
	4	C27H48N6NaO9	62.64	623.337498	-1.4	-0.9	28.4	6.5	even	ok
	5	C39H44N4NaO2	14.09	623.335647	-4.4	-2.8	34.1	19.5	even	ok

Figure S1. The HRESIMS spectrum of nocardamine.

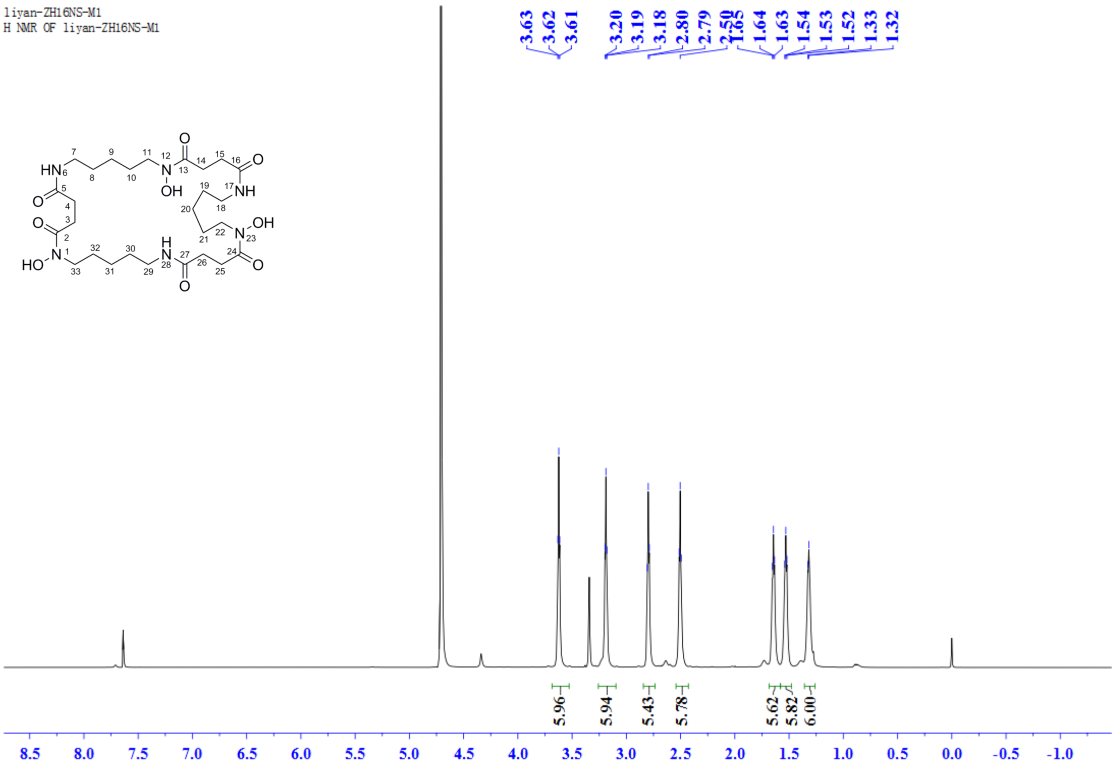

Figure S2. The ^{1}H NMR spectrum of nocardamine in $CDCl_3$/MeOD (1:1) at 750 MHz.

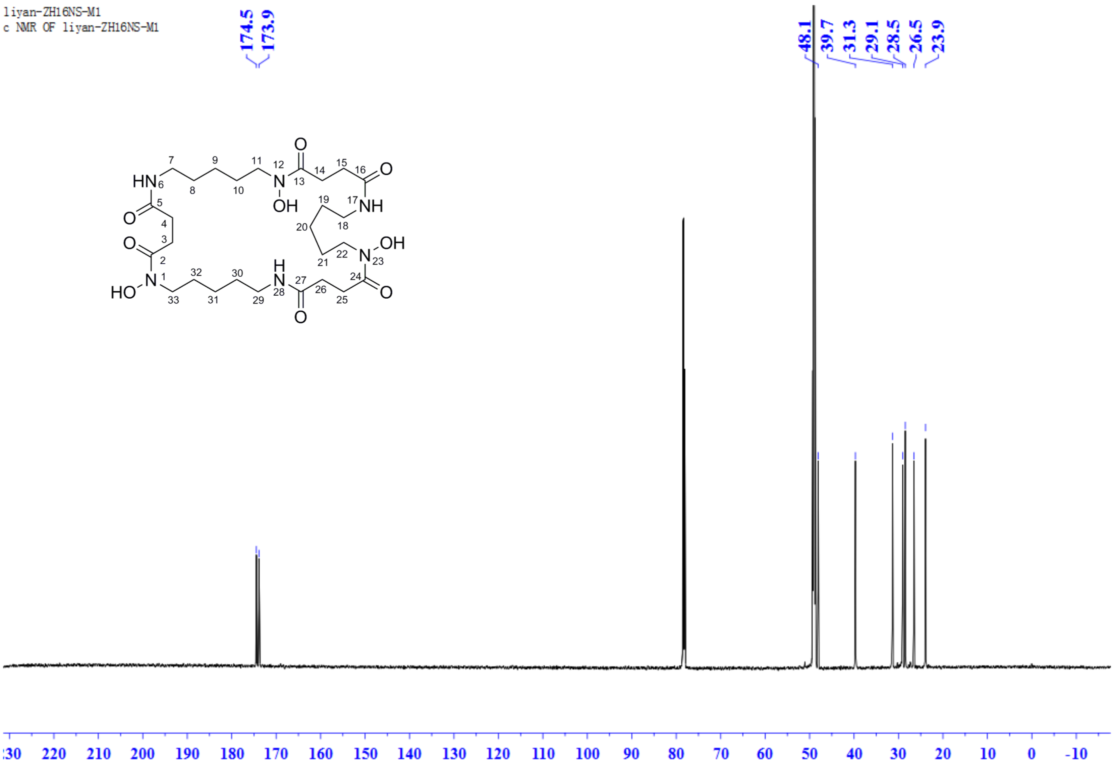

Figure S3. The ^{13}C NMR spectrum of nocardamine in $CDCl_3$/MeOD (1:1) at 175 MHz.

Table S1. Strains and plasmids were used and constructed in this study.

Strains /Plasmids	Description	Reference
E. coli		
DH5α	Host strain for cloning	invitrogen
BW25113/pIJ790	Host strain for PCR tatgeting	1
BW25113/ cosmid 113D1	Donor strain for conjugation	This study
ET12567/pUZ8002	Donor strain for conjugation	2
ET12567/pUB307	Assistant strain for conjugation	3
Streptomyces		
***S**treptomyces atratus* SCSIO ZH16	Wild-type strain	4
***S**treptomyces atratus* SCSIO ZH16S	*Streptomyces atratus* SCSIO ZH16 derivative where ilaS gene was in-frame deleted	This study
***S**treptomyces atratus* SCSIO ZH16NS	*Streptomyces atratus* SCSIO ZH16 derivative where *ilaN-ilaS* genes were in-frame deleted	This study
Plasmids		
pIJ773	the template for amplifying the Apr^R cassette	5
Cosmid 47H	genomic library cosmid of *S. atratus* SCSIO ZH16	This study
Cosmid 47H1	Cosmid 47H derivative where 8kb fragment were disrupted by insertional mutation with *aac(3)*Ⅳ	This study
Cosmid 47H2	Cosmid 47H1 derivative with the digestion by *Spe*I	This study
Cosmid 47H3	Cosmid 47H2 derivative where the *neo* gene was replaced with *aac(3)IV-oriT* cassette	This study

Table S2. Primers were used in this study.

Primers	Sequence
For inframe deletion mutant	
YilaN-*Spe*I-F	CTCCTGGCTGATGACCCGGCACGCCGACTGCACGGCGAT*ACTAGT*ATTCCG GGGATCCGTCGACC
YilaS-*Spe*I-R	CGACGCACTGCCCACACCCGACCACCCCAGCAACCGCCC*ACTAGT*TGTAG GCTGGAGCTGCTTC
YilaS-SpeI-F	GCACTTCAGCACAACGCTCTGATGTTTGAACTGCCG*ACTAGT*ATTCCGGGG ATCCGTCGACC
YilaS-speI-R	CACGTCGTTGAGCGCGGCCTCCAGCGAACCGGCGTCCAG*ACTAGT*TGTAG GCTGGAGCTGCTTC
For verification of inframe deletion mutant	
YIDilaN-F	AACACCGGAGACAGCCGT
YIDilaS-R	CCAACTGCTCCAACCCAG
YIDilaS-F	GCACCCGCTGATAAGGCTGTC
YIDilaS-R	CAGGTCCGTGAGGCGAGTCT
Clu11SVF	TCTGGATCGCGGGCGAGTC
Clu11SVR	GCTGTCGAGCTCGTCGAGG
For screen genome library	
Clu11SUF	GCTCGGTTTTCTGCCGGTCG
Clu11SUF	GTCGGTCGTCGTCTTGCGG
Clu11SDF	TGTTCCTGGCGTTCGTGGTC
Clu11SDF	GTTGTGCTGGTGCGGGTTGC

Table S3. General genome features of *S. atratus* SCSIO ZH16.

Features	Chromosome
Genome topology	linear
Assembly size (bp)	9,641,288
DNA-coding regions (bp)	8,407,203
CDS	9245
G + C content (%)	69.5%
Gene density (genes per kb)	0.958
tRNA genes	69
rRNA genes	18
Secondary metabolite gene clusters	26

Table S4. The gene clusters in *S. atratus* SCSIO ZH16.

Cluster No.	NP type	Size (Kb)	most similar known cluster	Similarity
Cluster 1	Nrps	118.0	Lipopeptide	22%
Cluster 2	Otherks-Nrps	98.9	Marformycins	12%
Cluster 3	T2pks-Terpene	65.4	Spore_pigment	83%
Cluster 4	Lantipeptide	75.7	Stenothricin	13%
Cluster 5	Phosphonate	40.9	Ansatrienin	7%
Cluster 6	T1pks-Nrps	54.7	Ilamycin	—
Cluster 7	Terpene	21.1	Steffimycin	19%
Cluster 8	Ectoine	10.4	Ectoine	100%
Cluster 9	Butyrolactone	11.1	γ-butyrolactone	100%
Cluster 10	T1pks	55.4	Tetronasin	3%
Cluster 11	Siderophore	11.8	Desferrioxamine B	100%
Cluster 12	T3pks	41.1	Naringenin	100%
Cluster 13	Nrps	88.9	Skyllamycin	48%
Cluster 14	Siderophore	14.7	Unknown	—
Cluster 15	Bacteriocin	11.3	Unknown	—
Cluster 16	Terpene	26.6	Hopene	84%
Cluster 17	Bacteriocin	12.6	Griselimycin	7%
Cluster 18	Other	44.1	Unknown	—
Cluster 19	Bacteriocin	10.8	Tautomycin	6%
Cluster 20	T1pks-Nrps	119.4	Herboxidiene	10%
Cluster 21	Terpene-Otherks-T1pks-Nrps	205.2	Vicenistatin	70%
Cluster 22	Terpene	59.0	Echosides	11%
Cluster 23	Nrps	63.8	Cyclomarin	13%
Cluster 24	Lantipeptide-Nrps	79.5	Streptolydigin	10%
Cluster 25	T1pks	46.4	Sporolide	8%
Cluster 26	Nrps	118.0	Enduracidin	16%

Supplementary References

1. Datsenko, K. A., Wanner, B. L. (2000) One-step inactivation of chromosomal genes in *Escherichia coli* k-12 using PCR products. P*roc. Natl. Acad. Sci. USA* 97, 6640-6645.

2. Macneil, D. J., Gewain, K. M., Ruby, C. L., Dezeny, G., Gibbons, P. H., Macneil, T. (1992) Analysis of *streptomyces avermitilis* genes required for avermectin biosynthesis utilizing a novel integration vector. *Gene* 111, 61-68.

3. Fiona Flett, Vassilios Mersinias, Colin P. Smith. (1997) High efficiency intergeneric conjugal transfer of plasmid DNA from *Escherichia coli* to methyl DNA-restricting *Streptomycetes*. *FEMS Microbiol. Lett.* 155, 223-229.

4. Ma, J.; Huang, H.; Xie, Y.; Liu, Z.; Zhao, J.; Zhang, C. (2017) Biosynthesis of ilamycins featuring unusual building blocks and engineered production of enhanced anti-tuberculosis agents. *Nat. Commun*. 8, 391.

5. Gust, B.; Challis, G. L.; Fowler, K.; Kieser, T.; Chater, K. F. (2003) PCR-targeted *Streptomyces* gene replacement identifies a protein domain needed for biosynthesis of the sesquiterpene soil odor geosmin. *Proc. Natl. Acad. Sci. USA* 100, 1541-1546.
